# Identification of bioactive components behind the antimicrobial activity of cow urine by peptide and metabolite profiling

**DOI:** 10.5713/ab.22.0249

**Published:** 2023-01-11

**Authors:** Rohit Kumar, Jai Kumar Kaushik, Ashok Kumar Mohanty, Sudarshan Kumar

**Affiliations:** 1 ICAR-National Dairy Research Institute, Cell Biology and Proteomics Lab, Animal Biotechnology Center (ABTC), Karnal, Haryana, 132001, India; 2 Current affiliation: ICAR-Indian Veterinary Research Institute, Nainital, Uttarakhand 263138, India

**Keywords:** Antimicrobial Peptides, Cow Urine, Mass Spectrometry, Metabolites, Solid-Phase Extraction

## Abstract

**Objective:**

Cow urine possesses several bioactive properties but the responsible components behind these bioactivities are still far from identified. In our study, we tried to identify the possible components behind the antimicrobial activity of cow urine by exploring the peptidome and metabolome.

**Methods:**

We extracted peptides from the urine of Sahiwal cows belonging to three different physiological states viz heifer, lactation, and pregnant, each group consisting of 10 different animals. The peptides were extracted using the solid phase extraction technique followed by further extraction using ethyl acetate. The antimicrobial activity of the aqueous extract was evaluated against different pathogenic strains like *Staphylococcus aureus*, *Escherichia coli*, and *Streptococcus agalactiae*. The safety of urinary aqueous extract was evaluated by hemolysis and cytotoxicity assay on the BuMEC cell line. The urinary peptides were further fractionated using high-performance liquid chromatography (HPLC) to identify the fraction(s) containing the antimicrobial activity. The HPLC fractions and ethyl acetate extract were analyzed using nLC-MS/MS for the identification of the peptides and metabolites.

**Results:**

A total of three fractions were identified with antimicrobial activity, and nLC-MS/MS analysis of fractions resulted in the identification of 511 sequences. While 46 compounds were identified in the metabolite profiling of organic extract. The urinary aqueous extract showed significant activity against *E. coli* as compared to *S. aureus* and *S. agalactiae* and was relatively safe against mammalian cells.

**Conclusion:**

The antimicrobial activity of cow urine is a consequence of the feeding habit. The metabolites of plant origin with several bioactivities are eliminated through urine and are responsible for their antimicrobial nature. Secondly, the plethora of peptides generated from the activity of endogenous proteases on protein shed from different parts of tissues also find their way to urine. Some of these sequences possess antimicrobial activity due to their amino acid composition.

## INTRODUCTION

Urine is the only biological fluid that possesses biomolecules originating from different parts of the body and hence, encapsulates the complete pathophysiology of the animal. A substantial part of this urine is constituted by peptides and proteins originating from the extracellular matrix, secreted exosomes, and tubular secretions [1,2]. In our earlier study, we reported thousands of peptide sequences in cow urine and their antimicrobial activity [2]. Apart from the peptide sequences originating from different protein sources as a result of proteolytic cleavage, some sequences in bovine are expressed and belong to a certain class of antimicrobial peptides viz. cathelicidin and defensins [3]. Limited literature is available on the identification of antimicrobial peptides in cow urine. One recent study established the constitutive expression of β-defensin-1 (DEFB1), β-defensin-4A (DFB4A), neutrophil defensin-1 (DEF1), neutrophil defensin-3 (DEF3) in cow urine [4]. In our previous work, we identified hundreds of possible antimicrobial peptide sequences in cow urine and currently in the process of the validation of their antimicrobial activity [2]. Studies have reported the antibacterial activity of cow urine against a wide spectrum of pathogenic bacteria such as *Salmonella typhi*, *Bacillus subtitlis*, *Bacillus cereus*, *Staphylococcus aureus*, *Staphylococcus epidermis*, *Pseudomonas aeruginosa*, *Proteus vulgaris*, *Escherichia coli*, *Pseudomonas fragi*, *Streptococcus agalactiae* etc. [5–7]. One study claimed that cow urine blocks the transfer of R-factor and prevent the development of antibacterial resistance in bacteria [8]. It is also shown to possess antifungal activity against *Fusarium oxysporum*, *Rhizoctonia solani*, *Sclerotium rolfsii*, *Fusarium lateritium*, clinical isolates of Candida species [9,10]. Besides antimicrobial activity, cow urine also shown to possess antineoplastic, immunomodulatory, and antioxidant activity [11,12]. Another study reported antibacterial, antifungal and anthelmintic activity of cow urine concentrate obtained after complete evaporation of cow urine. However, the components behind the activities were not determined [13].

In our recent work, we have profiled thousands of pep tides in different physiological states (Heifer, Lactation, and Pregnant) of the Sahiwal cow. Many of these sequences were functionally annotated to a certain bioactive group with the assistance of a web-based prediction platform. Sequences were categorized into four bioactivity classes: anticancer, antihypertensive, anti-inflammatory, and antimicrobial. Our current study is focused on pinpointing the presence of the activity in the fractions and simultaneously investigating the other factors responsible for the antimicrobial activity of cow urine.

## MATERIALS AND METHODS

### Ethical approval statement

The approval for the experiment was obtained from Institutional Animal Ethics Committee (IAEC) of National Dairy Research Institute (IAEC approval no. 41-IAEC-18-50). Methods were carried out in accordance with IAEC and ARRIVE guidelines. The urine samples were collected from the Sahiwal under the veterinary supervision. The animals are housed in the Livestock Research Centre (LRC) of National Dairy Research Institute (NDRI), Karnal. The Institute is governed by the Indian Council of Agricultural Research, Government of India. NDRI has all the necessary permits for the housing and care of animals for scientific purposes vide registration no. 1705/GO/ac/13/CPCSEA 3rd July, 2013 duly approved by Ministry of Environment and Forest, Govt. of India (Web site: http://envfor.nic.in).

### Sample collection and processing

The urine samples were collected from ten healthy females from each group Sahiwal heifers, Sahiwal lactation, and Sahiwal pregnant cows and processed as mentioned in our earlier study [2]. The sample for MS analysis was created by pooling and processed separately with three technical replicates. Fixed-time morning voids urine samples (approx 500 mL) were collected by massaging the perineum of the animal. The samples were transferred to the lab and filtered through a muslin cloth to remove any contaminating particulate matter followed by centrifugation at 7,000 rpm for 20 minutes to allow settlement of any cell debris or particulate matter. The microscopic examination was performed for individual samples, before and after the centrifugation, to observe the presence of red blood cells, white blood cells, pathogens and debris. The purified clean urine was further used for peptide extraction and purification.

### Peptide extraction

The clean urine samples were passed through ultra-filtration assembly (Thermo easyload Masterflex, model 7518-00; Thermo-Fisher Scientific, Barrington, IL, USA) with a 10 kDa molecular weight cut-off filter (Pall Minimate TFF Capsule), to separate the endogenous peptide mixture (PM). The pH of the obtained filtrate was adjusted to ≤3 using trifluoroacetic acid (TFA) for further treatment. The PM was subjected to manually prepared C-18 beads-based solid phase extraction columns (SPE). Briefly, the column was prepared using the C-18 reversed-phase silica gel (Cat. No. 60757-50G; Fluka Sigma-Aldrich, Buchs, Switzerland). A slurry of 20 grams of silica gel was prepared in methanol. Packing of the column was done by continuously stirring the slurry and then slowly draining it into the column followed by several washes with methanol. The packed column was conditioned using 90% methanol followed by equilibration with 10 column volumes of 90% methanol containing 0.1% TFA. After equilibration, PM was loaded with a flow rate of 0.5 mL/min followed by desalting using 5% methanol with 0.1% TFA. The desalted peptides were eluted by 60% acetonitrile (ACN) with 0.1% TFA. Processing of around 500 mL of urine yielded approx. A total of 30 to 50 mL of eluate with a dark brown appearance. For the removal of the dark brown substances (possible contaminating metabolites in urine) the ethyl acetate-based extraction was performed. The eluates were subjected to the double volume of ethyl acetate followed by end-to-end rotation for 5 min and allowed to settle for 10 min to differentiate into two layers. The upper organic layer was stored appropriately for metabolome profiling and the lower aqueous phase was aliquoted in 2 mL microcentrifuge tubes and dried by speedVac (Thermo savant ISS110 SpeedVac concentrator, ISS110-230; Thermo Fisher Scientific, Asheville, NC, USA).

The dried samples were stored at −80°C. Before the mass spectrometer run the routine sample cleanup was performed by Pierce C-18 Spin Columns as per the manufacturer’s protocol. The quantified samples were reconstituted in 20% ACN with 2% TFA (sample buffer, 3 μL for every 1 μL of sample). After loading and washing of the sample, peptides were eluted in 70% ACN with 0.1% TFA. The C18 eluates were extracted using ethyl acetate and the aqueous phase and organic phase were separated and stored for further analysis. Aqueous extracts from different animals were pooled and subjected to high-performance liquid chromatography (HPLC) fractionation.

### Metabolomics of ethyl acetate extract of Sahiwal cow urine

The C18 eluate obtained after SPE was extracted with ethyl acetate extract. The ethyl acetate was added to the C18 eluate in 1:2 and vortexed for 1 minute. Organic and aqueous layers were allowed to settle down. The upper organic layer was collected and pooled from all the animals groupwise. The antimicrobial activity of pooled organic layers was confirmed by a disc diffusion assay. To ascertain the possible reason behind the antimicrobial activity of organic or metabolite fraction, the sample was subjected to a liquid chromatography-mass spectrometry (LC-MS) run. Briefly, the pooled organic sample was lyophilized completely and desiccated to remove moisture. The powdered organic fraction was then subjected to LC-MS run for the identification of metabolites. All MS acquisitions were performed in the positive electrospray ionization mode. The capillary voltage, cone voltage, fragmentor voltage were 4 kV, 45 V, and 170 V, respectively. The gas temperature was set at 325 deg. The peptides were separated using micro-LC (Agilent 1260 binary LC System; Agilent Technologies, Santa Clara, CA, USA) through an analytical column (Agilent Zorbax Extend C18 RRHT column 50×2.1 mm, 1.8 μm) coupled with an ESI source (Bruker Daltonics, Bremen, Germany) 6540 ultra-high-definition accurate mass quadrupole time of flight (qTOF) mass spectrometer. The elution was performed with a flow rate of 300 μL/min and a continuous gradient of 5% to 95% ACN over 30 min. In the solvent system, Solvent A was 100% water with 0.1% formic acid, and solvent B was 100% ACN with 0.1% formic acid. Data were acquired at a scan rate of 3 Hz in the mass range of 100 to 100 m/z. Further data was analyzed with Mass hunter qualitative software and the METLIN database.

### Disc diffusion assay

Disc diffusion assay was performed as per CLSI guidelines. The aqueous phase containing urinary peptides was resuspended in 100 μL Milli-Q water to assess the antimicrobial activity of the peptides by disc diffusion assay. The volume obtained after processing and C18-based solid-phase extraction constitute 4% to 6% (20 to 30 mL approx.) of total urine volume (400 to 500 mL). Post SPE, the urinary peptides were concentrated to 4% to 6% of the total urine volume. A volume of 1 mL of the SPE eluate was aliquoted into 1.5 mL microcentrifuge tubes and lyophilized in a speed vac. The lyophilized extract was reconstituted in 200 μL of Milli-Q water and 30 μL of the extract was coated onto 6 mm sterile discs (SD067-1VL; HIMEDIA, Mumbai, Maharashtra, India). The urinary aqueous extract volume was concentrated almost 80 times before antimicrobial activity determination. The discs were allowed to dry under a laminar hood. A lawn of 0.5 McFarland equivalents of test cultures (*Staphylococcus aureus* ATCC 29213, *Escherichia coli* ATCC 25922, and *Streptococcus agalactiae* ATCC 27956) were swabbed on surface of Mueller Hinton Agar (GM173-500G; HIMEDIA, India) and allowed to dry. With the help of sterile forceps, the coated discs were placed on the lawn of the test culture and incubated overnight. Bovine serum albumin digest was used as a negative control in the experiment. The appearance of the inhibition zone confirms the antimicrobial activity of the buffalo or cow urinary peptides.

### MIC determination

A modified form of broth microdilution assay was performed, The MIC was determined using Resazurin dye-based broth microdilution assay. The aqueous extract containing total urinary peptides obtained from buffalo urine was weighed and dissolved in Mueller Hinton broth (MHB). A 100 μL of peptide solution was dispensed in each well of column 1 and 50 μL of MHB was dispensed in columns 2 to 9. Using a multichannel pipette, peptides from column 1 were double serially diluted in columns 2 to 9, resulting in 50 μL of peptides’ solution per well. Column 11 containing 100 μL of standardized inoculums was taken as growth control for the experiment. Inoculums for the experiment were prepared by direct suspension of isolated colonies in normal saline from a 24-hour agar plate and the turbidity of the suspension was adjusted to 0.5 McFarland indicator. The adjusted suspensions were then diluted by 1:20 in MHB yielding approx. 5×10^5^ CFU/mL. A total of 5 μL of the test culture inoculums were dispensed in wells of columns 1 to 9 and the growth control column. The inoculums of the test cultures were prepared and dispensed within 15 minutes. The plate was sealed and incubated for 24 hours at 37°C. Using a multichannel pipette, 20 μL of the Resazurin dye was added to each well at 0.2 mg/mL concentration and then incubated for another 2 to 4 hours. After incubation, the wells with slight color change were scored as MIC.


$$\%\;\text{Bacterial viability}=({\text{OD}}_{\text{Treated}}-{\text{OD}}_{\text{negative control}})/({\text{OD}}_{\text{positive control}}-{\text{OD}}_{\text{negative control}})\times 100$$

### Kill kinetics assay

Kill kinetics were determined for cow urinary aqueous extract after confirming the antimicrobial activity. Kill kinetics were performed at three different concentrations of peptides and total urinary peptides containing aqueous extract viz. 0.5×MIC, 1×MIC, 2×MIC along with growth control and sterility control. Treatment was given at 0 hour timepoint. Samples were taken out, serially diluted at 2, 4-, 8-, 12-, and 24-hour time intervals, and drop plated on an agar plate to count colony forming unit post 16 to 18 hours incubation.

### HPLC based fractionation of cow urinary peptides

Pooled urinary peptides from the heifer group of Sahiwal cows were subjected to fractionation to determine the fraction possessing antimicrobial activity. Twenty fractions were collected from 12 HPLC runs. The corresponding fractions from twelve runs were pooled and lyophilized. For determination of the antimicrobial activity of the fractions, a lawn of 0.5 McFarland equivalent of test cultures (*Staphylococcus aureus* ATCC 29213) was swabbed on the surface of Mueller Hinton Agar (GM173-500G; HIMEDIA, India) and allowed to dry. Lyophilized fractions were reconstituted in 60 μL of Mili Q water, out of which 20 μL was coated on 6 mm sterile discs and allowed to dry under the laminar hood. With the help of sterile forceps, the coated discs were placed on the lawn of the test culture and incubated overnight. Fractions exhibiting antimicrobial activity were pooled and subjected to nLC-MS/MS for peptide sequence identification.

### Electrospray ionization tandem mass spectrometry LC-MS/MS analysis

The reconstituted peptides were used for shotgun proteomics experiments for the identification of endogenous peptides. The peptides were separated using micro-LC (Thermo UltiMate 3000 HPLC System; Dionex, Sunnyvale, CA, USA) through an analytical column (Supelco Sigma-Aldrich, St. Louis, MO, USA; Ascentis Express C18, 25 cm×4.6 mm, 2.7 μm) coupled with ESI source (Bruker Daltonics, Germany) spray in Maxis-HD qTOF (Bruker, Germany) mass spectrometer. The elution was performed with a flow rate of 150 μL/min and a continuous gradient of 5% to 75% ACN over 135 min. In the solvent system, solvent A was 100% water with 0.1% formic acid, and solvent B was 100% ACN with 0.1% formic acid. Data were acquired in the data-dependent mode in a mass spectrometer that operated automatically switching between MS and MS/MS acquisition. The precursor ion MS spectra scan range of 200 to 2,200 (m/z) was used in the Q-TOF with resolution R = 75,000. The six most abundant precursor ions were searched for detection of different masses during acquisition and selected for fragmentation using collision-induced dissociation with a fixed cycle time of 3 s along with 2 min of release for exclusion filter (Q-TOF processing software; Bruker Daltonics, Germany).

### Data processing

The .d format raw data files were subjected to the TPP pipeline for the identification of endogenous peptides. For analysis, the otof generated raw (.d) files were converted to mzML format using MSconvert GUI using the default parameters. The converted mzML files were searched for MS/MS spectra using the Trans-Proteomic Pipeline version 5.1.0 released on 2017-11-03 on in-house combined UniProt Bos taurus (cow), Bubalus bubalis (buffalo), and common contaminant sequences, and an equal number of decoy sequences database. Briefly, for the analysis, the peptide assignments were performed using Comet. The search parameters included un-digested peptides and the remaining parameters were kept as default. The minimum peptide length parameter was set to 6 amino acid residues. Further Peptide Prophet and Protein Prophet algorithms were used in the pipeline to compute the probabilities score for both individually searched peptides and the respective proteins. The accurate mass model in Peptide Prophet was used for high confidence peptide identifications to boost the probability of peptide identification. Another protein validation step was executed using both Peptide Prophet and Protein Prophet scores, where the protein was authenticated if it contained a minimum of two top-ranked peptides with each peptide probability score above 95%. All the search engine results were merged and validated using iProphet. This method takes the input of Peptide Prophet spectrum-level results from multiple LC-MS/MS runs and then computes a new probability at the level of a unique peptide sequence (or protein sequence). This framework allows for the combination of results from multiple search tools and takes into account other supporting factors, including the number of sibling experiments identifying the same peptide ions, the number of replicate ion identifications, sibling ions, and sibling modification states. A model of iProphet performance concerning the number of correct identifications versus error. An iProphet probability of more than 0.95 was used as the cutoff for the final identification of the protein.

### Bioinformatic analysis

Antimicrobial peptides were predicted using CAMPR3 server. Sequence logo of 10 amino acid residues from the N-terminal was prepared using weblogo.

### Haemolysis assay

For haemolytic assay, briefly, 10 mL of venous blood was drawn from the human donor directly into the ethylenediaminetetraacetic acid (K2-EDTA)-coated vacutainer tubes. Blood was centrifuged at 500×g for 5 minutes and level of hematocrit and plasma was marked on the tube. Plasma was aspirated out and discarded into biohazardous waste. The hematocrit tube was filled with 150 mM NaCl solution up to the marked level of plasma. The tube was gently inverted a few times to ensure proper mixing and then centrifuged at 500×g for 5 minutes. The washing step was repeated, and the supernatant was replaced with phosphate-buffered saline (PBS) (pH 7.4). A 1:50 dilution of erythrocytes was prepared by adding 1 mL of erythrocytes to 49 mL of PBS. A haemolysis experiment was performed on a 96-well plate. Aqueous extract stock was diluted in 190 μL of erythrocytes to obtain final test concentrations of 4,000, 2,000, 1,000, 500, 250, 125 μg/mL. To the positive control wells, 10 μL of 20% Triton X-100 was added in positive control wells and 10 μL of PBS was added to negative control wells. For each peptide and control, samples were loaded in triplicate. Using a multichannel pipette 190 μL of homogenous erythrocytes was added to each well. The plate was incubated at 37°C for one hour on an orbital shaker. The plate was centrifuged at 500×g for 5 minutes to pellet non-lysed erythrocytes. For absorbance measurement, a 100 μL of supernatant was transferred into clear flat bottom 96 well plate. The absorbance of the supernatant was measured by the Tecan nanoquant 96 well plate reader. The % haemolysis was calculated by the following formula:


$$({\text{OD}}_{\text{test}}-{\text{OD}}_{\text{neg}})/({\text{OD}}_{\text{pos}}-{\text{OD}}_{\text{neg}})\times 100$$

### Cytotoxicity assay

Cytotoxicity assay was performed as described in the manufacturer’s protocol (abcam MTT assay kit: ab211091; abcam, Cambridge, UK). BuMEC cells were trypsinized and seeded in 96 well plate at a concentration of 1×10^4^ cells/well in a 100 μL culture medium. The plates were incubated for 24 hours at 37°C under 5% CO_2_ to obtain an even number of cells in all the wells. After 24 hours of incubation, the old medium was removed from each well carefully without disturbing the monolayer. A dilution series of buffalo urinary peptides were prepared in a different 96 well plate to obtain the final test concentrations of 5,000, 2,500, 1,250, 625, 312.5, 156.25 μg/mL. For cell death positive controls, a 100 μL of 10% dimethyl sulfoxide (DMSO) was dispensed into positive control wells, and 100 μL of culture medium was added to negative control wells. The experiment was performed in triplicate. The urinary peptide dilution was then dispensed into designated wells in 96-well culture plate and incubated for another 24 hours at 37°C under 5% CO_2_. Post 24 hours incubation, culture medium containing test concentration of urinary peptide was replaced with a fresh culture medium. A 20 μL of MTT reagent at final concentration of 5 mg/mL was added into each well and incubated for 3 hours at 37°C in the CO_2_ incubator. The MTT solution was removed carefully without disturbing the formazan crystal, followed by the addition of 150 μL of DMSO to each well and the plate was agitated on an orbital shaker for 15 minutes to solubilize the formazan crystals. The absorbance was measured by Tecan nanoquant 96 well plate reader at a wavelength of 565 nm with a reference wavelength of 620 nm. Percent BuMEC inhibition was calculated using following formula:


$$\begin{array}{l} \%\;\text{BuMEC survival}=({\text{OD}}_{\text{Treated}}-{\text{OD}}_{\text{Blank}})/({\text{OD}}_{\text{Untreated}}-{\text{OD}}_{\text{Blank}})\times 100\\ \%\;\text{BuMEC inhibition}=100-\%\;\text{BuMEC survival} \end{array}$$

## RESULTS

### Antimicrobial activity of the aqueous extract

**Figure 1:**
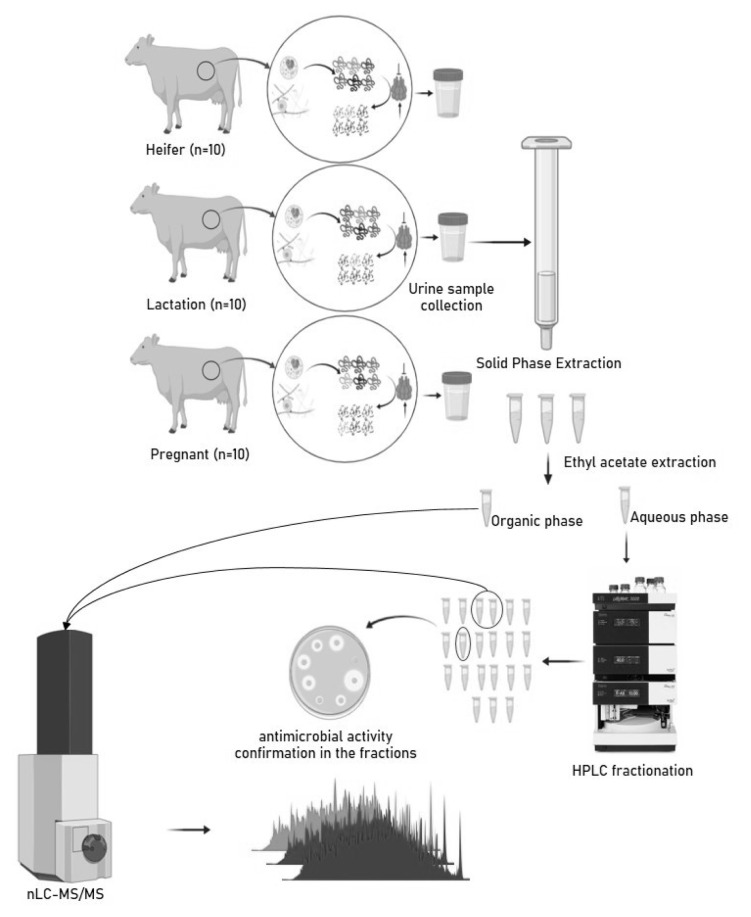
Experimental design: collection, processing, extraction of peptides and metabolites, and analysis using nLC-MS/MS.

**Figure 2:**
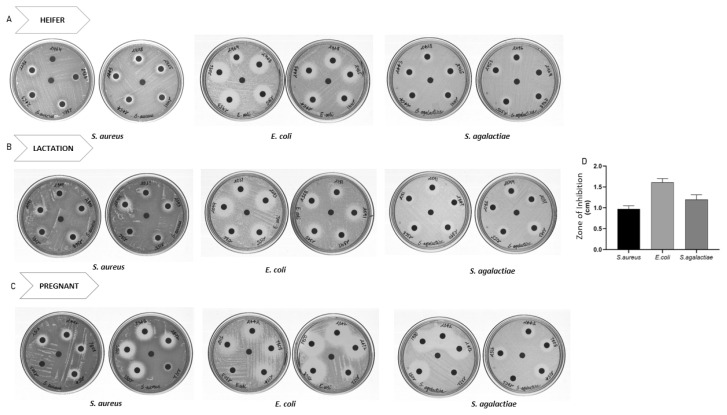
Disc diffusion assay of urinary peptides containing aqueous extract from different animals belonging to physiological groups: (A) heifer (B) lactation and (C) Pregnant. Urinary peptide from 30 Sahiwal cow were coated onto discs and Disc diffusion assay was performed against *S. aureus*, *E. coli* and *S. agalactiae*. (D) Diameter of zone of inhibition observed against *S. aureus*, *E. coli* and *S. agalactiae*. The bar and error bar in the figure represent mean±standard error of mean.

**Figure 3:**
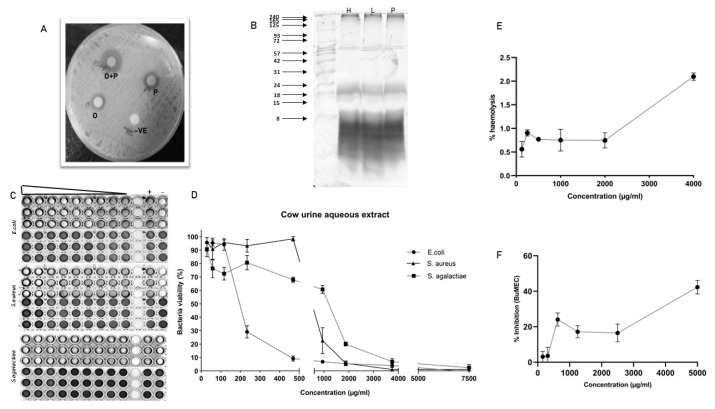
Characterization of antimicrobial activity of urinary peptide: (A) Antimicrobial activity of different phases obtained after ethyl acetate extraction; O+P: C18 eluate, P: urinary peptide-containing aqueous extract, O: metabolite containing organic phase. (B) Urinary peptide visualization from different physiological groups using Tricine-SDS-PAGE. Ladder shown in the image ranges from 8 to 240 kDa (C) Resazurin-based broth microdilution assay for MIC determination. The well with blue color indicates the dead bacterial population and pink color indicates the well with viable bacterial population. The ‘+’ sign indicates the well with bacterial growth (positive control) whereas ‘−’ sign are well without bacterial inoculation (negative control or sterility control) (D) bacterial survival rates in a dose-dependent manner. (E) Haemolysis assay at different concentrations of urinary aqueous extract. (F) Cytotoxicity assay against BuMEC cell line (Buffalo mammary epithelial cells) at different concentrations of urinary aqueous extract. Experiments were performed in triplicate and the points on the line graph represent mean±standard error of the mean. SDS-PAGE, sodium dodecyl sulfate-polyacrylamide gel electrophoresis.

The peptide containing aqueous extract for the analysis was obtained as described in the Figure 1. The antimicrobial activity of urinary peptides isolated from 30 different animals belonging to different physiological groups was determined using disc diffusion method (Figure 2). The antimicrobial activity of different phases obtained after ethyl acetate extract of C18 eluate was confirmed. Both organic and aqueous extracts showed the development of a zone of inhibition against the pathogenic bacteria (Figure 3A). Before determining antimicrobial activity, a tricine-sodium dodecyl sulfate-polyacrylamide gel electrophoresis (SDS-PAGE) was run to confirm the presence of peptides (Figure 3B). The zone of inhibition developed against *E. coli* was significantly higher as compared to *S. aureus* and *S. agalactiae*. Due to variability in the zone of inhibition, the aqueous extracts from different animals were pooled to determine the minimum inhibitory concentration. The MICs of the aqueous extract against *E. coli*, *S. aureus*, and *S. agalactiae* were 0.468 mg/mL, 3.75 mg/mL, and 3.75 mg/mL respectively (Figure 3C and 3D).

### Haemolysis and cytotoxicity assay

A dose-dependent increase in haemolysis was observed but it was not significant. The highest haemolysis observed was 2% at a 4 mg/mL concentration. Cytotoxicity assay against BuMEC cell line showed almost 40% cell inhibition at 4 mg/mL concentration (Figure 3E and 3F).

### Kill kinetics

**Figure 4:**
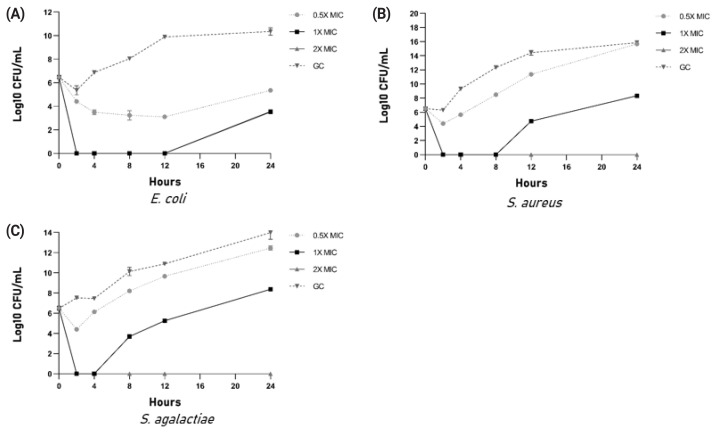
Kill kinetics study at three different concentrations viz. 0.5×, 1×, and 2× MIC against: (A) *Escherichia coli*, (B) *Staphylococcus aureus*, and (C) *Streptococcus agalactiae*. Experiments were performed in triplicate and the points on the line graph represent mean± standard error of the mean.

Kill kinetics studies showed that 2×MIC concentration in all three bacterial species completely inhibited growth. In the case of 1×MIC, growth resumption was observed in all three bacteria (Figure 4A–4C).

### Determination of the antimicrobial activity of HPLC fractions

**Figure 5:**
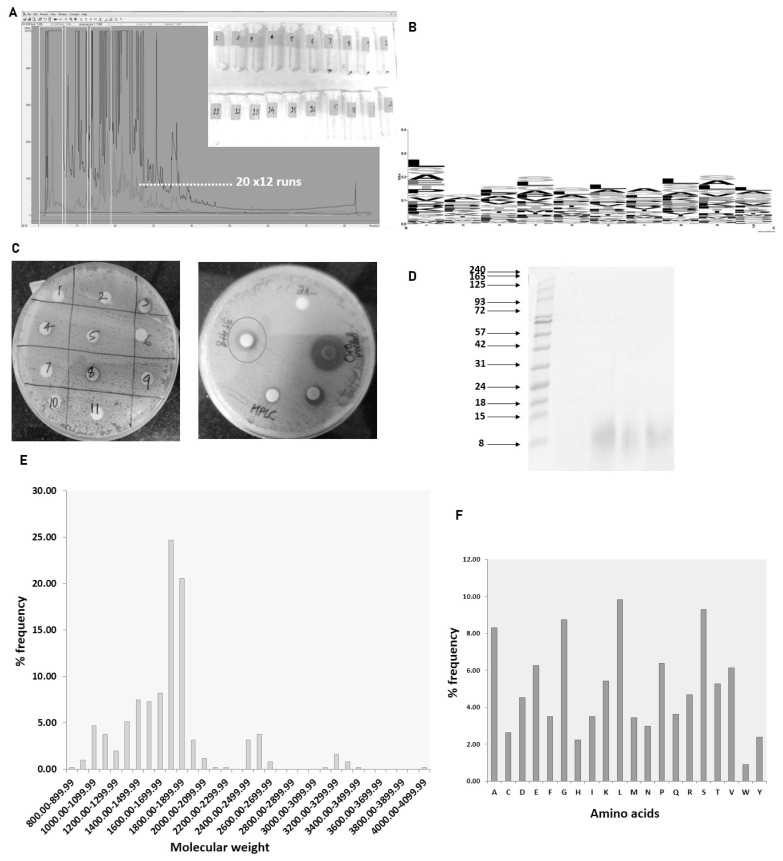
Antimicrobial activity of urinary peptide fractions obtained from C18 and characterization: (A) HPLC-based fractionation of pooled aqueous extract from different animals. (B) Sequence logo generation of predicted antimicrobial sequences. (C) Antimicrobial activity determination of different fractions. (D) Tricine SDS-PAGE of peptides in fractions 3, 4, and 8 of urinary peptides. Frequency distribution: (E) molecular weight of peptide (F) amino acids present in fractions 3, 4, and 8. HPLC, high performance liquid chromatography; SDS-PAGE, sodium dodecyl sulfate-polyacrylamide gel electrophoresis.

The antimicrobial activity of different fractions of cow urinary aqueous extract was determined by a disc diffusion assay. Fractions 3, 4, and 8 showed antimicrobial activity against *S. aureus* (Figure 5C). The developed zone of inhibition was very feeble for the individual fractions. So, to ascertain the presence of antimicrobial activity, the fractions were pooled. The zone of inhibition developed for pooled fractions was significant and each fraction was run in tricine-SDS-PAGE to visualize the peptide (Figure 5D). The fractions with antimicrobial activity were subjected to nLC-MS/MS, identifying 511 peptide sequence in the pooled fractions. Analysis of retrieved sequences showed the abundance of peptides in the molecular weight range of 1.4 to 1.9 kDa (Figure 5E). The amino acid profile showed the dominant presence of Ala, Gly, Leu, Pro, and Ser residues (Figure 5F). These sequences were then used for the prediction of antimicrobial peptides.

### Antimicrobial peptide prediction and sequence logo

The 511 sequence obtained from nLC-MS/MS run was subjected CAMPR3 antimicrobial peptide prediction tool. We identified 28 potential antimicrobial high scorings (>0.9) sequences using the Support Vector Machine algorithm (Table 1). The N terminal 10 amino acids from the predicted sequences were picked to deduce the compositional biases in the antimicrobial sequences. Amino acids like Leu, Gly, Ser, and Ala showed a dominant presence in these positions (Figure 5B).

### Metabolite profile of ethyl acetate extract

Our study identified a total of 46 compounds in the ethyl acetate extract of C18 eluate. Most of these compounds have plant origins. The identified compounds’ functional activity was retrieved from PubChem and literature searches (Table 2).

## DISCUSSION

Many works of literature have discussed the antimicrobial activity of cow urine but the information about the compound or the type of compounds responsible for the activity is lacking. Our work demonstrated that the activity of cow urine can be attributed to the presence of diverse peptide sequences and metabolites from plant origin. In our study, we confirmed the antimicrobial activity in the ethyl acetate extract and peptide fraction described here as an aqueous extract. The antimicrobial activity of the aqueous extract was confirmed in a large group of animals (n = 30) against *S. aureus*, *E. coli*, and *S. agalactiae*. The aqueous extract showed significant antimicrobial activity against *E. coli* followed by *S. aureus* and *S. agalactiae*. However, there was variability in the size of the zone suggesting that the activity varies from animal to animal. Therefore, we pooled the aqueous extract to determine the MIC, which indicated its enhanced activity toward *E. coli*. The graph indicates the steep decline in the *E. coli* population after 200 μg/mL concentration. We did not find any significant difference in the activity against *S. aureus* and *S. agalactiae*. The kill kinetics studies showed that aqueous extract was able to completely inhibit bacterial growth at 2× MIC concentration. The assessment of the haemolytic index and cytotoxicity of the extract showed its selectivity toward bacterial populations and certainly not to mammalian cells. Summarily, the urinary peptides containing aqueous extract have bactericidal activity and were selective to the bacterial population. Comparatively, it showed less inhibitory activity against the mammalian cell line (BuMEC).

The presence of antimicrobial activity in the fractions in trigued us to inspect the sequence information using the mass spectrometry technique. The molecular characteristics of the peptide sequences can reveal a plethora of information. In our data, peptides with a molecular weight range of 1.7 to 1.8 kDa were predominately present in the fraction. In line with our previous study on total urinary peptidome, amino acids such as Ala, Gly, Leu, Pro, and Ser constituted the major portion of the identified peptides. Total urinary peptides visualized using tricine-SDS-PAGE showed the presence of small molecular weight peptides (Figure 3B). The amino acid composition of peptides is the determinant of bioactive properties. Various studies correlated the amino acid compositional biases of the peptide to a certain activity. One study suggested that antimicrobial peptides are mostly dominated by the Arg, Lys, Leu, and Gly residues [14]. Most of the AMPs are cationic and selectively target the bacterial membrane rich in anionic phospholipids, which are absent in mammalian cells [15]. The net positive charge assists in the interaction with the negatively charged bacterial membrane while hydrophobic residues facilitate the penetration of peptides into the membrane [16,17]. The predicted AMPs in our data showed the predominant presence of Leu and Ser residues.

Two compounds identified in ethyl acetate extract are previously reported with antimicrobial activity. Compounds like phlorizin chalcone and baicalin are reported to possess antimicrobial activity [18–20]. Baicalin can be considered the compound with the most prolific activities against varieties of conditions. The compound is derived from *Scutellaria baicalensis*, *Scutellaria amoena*, *Thalictrum baicalense*, etc. Diverse activities like ferroptosis inhibitor, neuroprotective, antineoplastic, cardioprotective, antiatherosclerotic, antioxidant, RNA-directed RNA polymerase inhibitor, and anticoronaviral are also associated with the baicalin [21–28]. Phlorizin chalcone is a member of flavonoids and is found in the *Eucalyptus species*, *Malus* species, and various other species [29]. The compound’s preventive role has been extensively studied in the context of hyperglycemia and insulin resistance. Studies have reported the activity of compounds can counter the rise in the blood glucose level and can overcome the condition of insulin resistance [30,31]. The metabolite also possesses antioxidant and membrane permeabilizing activity [32,33]. Deoxyloganin, a methyl ester, and a beta-D-glucoside is a derivative of loganin found in *Strychnos nux-vomica* and *Verbena officinalis*. The metabolite has a preventive activity against diabetic nephropathy and a hepatic protective activity [34,35]. Isoliquirtin, one of the plant-derived metabolites has also antineoplastic activity [36,37]. Till date, we have not found any literature mentioning the presence of plant-based metabolites in cow urine. So, the bioactive peptides are not solely responsible for the activity of cow urine but also several plant-derived metabolites which exerts a wide spectrum of activities.

## CONCLUSION

In our present study, we tried to investigate the components behind the antimicrobial activity of cow urine. The study indicated the two possible components behind the antimicrobial activity of cow urine viz. antimicrobial peptides and plant origin metabolites. Antimicrobial peptides are present in every stratum of life starting from the simplest life form to the complex. These sequences provide immunity to the host against a broad spectrum of pathogenic threats. Our findings suggest that there is a pool of antimicrobial sequences in endogenous peptides originating from the animal body by the action of proteases. These sequences exhibit antimicrobial activity due to their amino acid composition and physicochemical properties. The other component is metabolites emanating from feed sources, some of the metabolites are bioactive and display a wide array of activities including antimicrobial activity. The activities of the identified metabolites have been validated in dozens of studies but the antimicrobial activity of peptide sequences needs to be functionally validated.

## Figures and Tables

**Table 1:** List of antimicrobial peptide sequences predicted using CAMPR3

S. no	Peptides	Prediction	SVM score
1	VGGGCCPCPCPP	AMP	0.917
2	CQPSCSTSSPCHTSCF	AMP	0.949
3	CQPSCSTSSPCHTSCF	AMP	0.949
4	LVNKKWMLALLR	AMP	0.957
5	GIPGIIGGIAGAVTASIANL	AMP	0.966
6	GLGSKKHPLHLLL	AMP	0.928
7	CIVKIPLTKMKTMQ	AMP	0.982
8	FAKLPKGLKHLNLSK	AMP	0.959
9	FVIIFLGVVLVMKKR	AMP	0.951
10	YCHRICYHPTCCC	AMP	0.997
11	GATKFGSQASQKFWGS	AMP	0.91
12	DEEPPTEQDKRKKML	AMP	0.971
13	VVEKIEDLLQSEENK	AMP	0.973
14	PYDSSEDDKEYVG	AMP	0.998
15	KRKKKKKDKDKAKLL	AMP	1
16	SNDDSSSYDK	AMP	0.999
17	VAKLRASAVLGFAVGT	AMP	0.913
18	GKADGNIKKQKKVR	AMP	0.937
19	ACSSCQRSIVGVRYQCSLCPS	AMP	0.941
20	LIKTILRLPLAQQRKKAF	AMP	0.943
21	DSSSYFCSAGDT	AMP	0.99
22	EEEEEDEDDNM	AMP	1
23	VAVAKIASHVVKN	AMP	0.944
24	LALFVVSFSQVLLLKS	AMP	0.901
25	SGSSECMWCSNMKQC	AMP	0.978
26	LVVKLLVKLNGQLM	AMP	0.952
27	LVRAGLGLDSNALKKLK	AMP	0.945
28	QALWMLLLKGKHIKKLK	AMP	0.982

**Table 2:** List of metabolites identified in the ethyl acetate extract of C18 eluate

S. no.	Compound label	Name	m/z	Retention time	Mass
1	Cpd 5: 2,5-Diamino-6-(5-phospho-D-ribitylamino)pyrimidin-4(3H)-one	2,5-Diamino-6-(5-phospho-D-ribitylamino)pyrimidin-4(3H)-one	356.0976	1.63	355.0905
2	Cpd 24: Melampodinin	Melampodinin	523.18	2.98	522.1724
3	Cpd 48: Trifloxystrobin	Trifloxystrobin	409.1374	4.72	408.13
4	Cpd 94: Demethylalangiside	Demethylalangiside	492.1862	5.77	491.1787
5	Cpd 102: Syringin	Syringin	373.1494	5.82	372.1421
6	Cpd 133: Deoxyloganin	Deoxyloganin	397.1475	6.1	374.1583
7	Cpd 139: Tuberonic acid glucoside	Tuberonic acid glucoside	389.1825	6.18	388.1752
8	Cpd 160: Syringin	Syringin	373.1498	6.43	372.1426
9	Cpd 162: SN38 glucuronide	SN38 glucuronide	569.1777	6.47	568.1699
10	Cpd 182: 2-(Biaryl)carbapenems	2-(Biaryl)carbapenems	350.1387	6.64	349.1315
11	Cpd 208: Trovafloxacin	Trovafloxacin	417.1174	6.91	416.1102
12	Cpd 210: Phlorizin chalcone	Phlorizin chalcone	435.1282	6.91	434.1207
13	Cpd 218: Baicalin	Baicalin	447.0921	7.09	446.0848
14	Cpd 255: Isoliquiritin	Isoliquiritin	419.1334	7.58	418.1261
15	Cpd 257: Austrobailignan 1	Austrobailignan 1	383.1123	7.58	382.105
16	Cpd 262: Luteolin 7-O-glucuronide	Luteolin 7-O-glucuronide	463.0873	7.67	462.0799
17	Cpd 269: Lusitanicoside	Lusitanicoside	443.1914	7.75	442.1839
18	Cpd 279: Isopentenyladenine-9-N-glucoside	Isopentenyladenine-9-N-glucoside	364.1969	7.85	363.1896
19	Cpd 281: Demethylalangiside	Demethylalangiside	492.1863	7.89	491.179
20	Cpd 312: Nigakilactone M	Nigakilactone M	395.2062	8.28	394.1988
21	Cpd 339: Rehmaionoside A	Rehmaionoside A	413.2175	8.38	390.2282
22	Cpd 344: Portulacaxanthin II	Portulacaxanthin II	375.1187	8.39	374.1115
23	Cpd 353: Dihydroergotamine	Dihydroergotamine	584.2904	8.44	583.2828
24	Cpd 365: Rehmaionoside A	Rehmaionoside A	391.233	8.5	390.2255
25	Cpd 402: 10-Deacetyl-2-debenzoylbaccatin III	10-Deacetyl-2-debenzoylbaccatin III	441.2116	8.85	440.2042
26	Cpd 445: Buspirone	Buspirone	386.2541	9.21	385.2468
27	Cpd 446: Tetrahydroaldosterone-3-glucuronide	Tetrahydroaldosterone-3-glucuronide	541.2651	9.22	540.2577
28	Cpd 458: Ophiopogonin D	Ophiopogonin D	855.4701	9.26	854.4626
29	Cpd 464: Aurantio-obtusin beta-D-glucoside	Aurantio-obtusin beta-D-glucoside	493.1341	9.37	492.1268
30	Cpd 480: Obacunone	Obacunone	455.2056	9.52	454.1978
31	Cpd 488: Myriocin	Myriocin	402.2858	9.6	401.2784
32	Cpd 560: Pentacarboxyl porphyrinogen III	Pentacarboxyl porphyrinogen III	701.2802	10.28	700.2727
33	Cpd 577: Glycocholic Acid	Glycocholic Acid	466.3161	10.51	465.3085
34	Cpd 600: Ergosine	Ergosine	548.2856	10.72	547.278
35	Cpd 613: Clivoline	Clivoline	406.1862	10.91	405.1788
36	Cpd 617: beta-Peltatin A methyl ether	beta-Peltatin A methyl ether	429.1544	10.95	428.1471
37	Cpd 625: Aureothin	Aureothin	398.1592	11.1	397.152
38	Cpd 628: Z-Phe-Phe-CHN2	Z-Phe-Phe-CHN2	400.2262	11.14	399.2189
39	Cpd 669: L-Urobilinogen	L-Urobilinogen	597.3632	11.75	596.3556
40	Cpd 692: Picrasin C	Picrasin C	423.2353	12.14	422.2279
41	Cpd 704: Diterpenoid EF-D	Diterpenoid EF-D	475.2695	12.68	474.2623
42	Cpd 709: Eplerenone	Eplerenone	415.212	12.82	414.2048
43	Cpd 722: 1,2-Di-(9Z,12Z,15Z-octadecatrienoyl)-3-(Galactosyl-alpha-1-6-Galactosyl-beta-1)-glycerol	1,2-Di-(9Z,12Z,15Z-octadecatrienoyl)-3-(Galactosyl-alpha-1-6-Galactosyl-beta-1)-glycerol	469.2963	13.06	936.578
44	Cpd 789: Tacrolimus	Tacrolimus	804.4875	15.26	803.4803
45	Cpd 823: Stigmatellin Y	Stigmatellin Y	485.2901	17.15	484.2823
46	Cpd 833: Diisooctyl phthalate	Diisooctyl phthalate	391.285	20.9	390.2776
